# Innovative approaches to treatment - refractory depression: The ketamine story

**DOI:** 10.4103/0019-5545.64573

**Published:** 2010

**Authors:** T. S. Sathyanarayana Rao, Chittaranjan Andrade

**Affiliations:** Department of Psychiatry, JSS University, JSS Medical College Hospital, Mysore - 570 004, India; 1 Department of Psychopharmacology, National Institute of Mental Health and Neurosciences, Bangalore - 560 029, India

Antidepressant drugs have traditionally addressed the monoamine triumvirate: serotonin, noradrenaline, and dopamine. There have been predominantly two mechanisms recruited in drug action - inhibition of monoamine reuptake and inhibition of intravesicular monoamine catabolism. Tianeptine, which *increases* the synaptic reuptake of serotonin, and mirtazapine, which blocks alpha-2 adrenergic autoreceptors and heteroreceptors, are examples of the very few drugs which have other mechanisms of action. Yet, even these two antidepressants target the traditional neurotransmitters in psychiatry.[1]

Recent evidence suggests that glutamate-mediated neuroplasticity may be the final common pathway of antidepressant action;[2,3] tianeptine is the best-studied antidepressant in this regard.[4] If glutamate holds the key to recovery from depression, then treatments that directly address glutamatergic neurotransmission may exhibit greater antidepressant efficiency.

Glutamate acts on metabotropic receptors and ionotropic receptors in the brain. Ionotropic receptors surround ion channels and are of three types: N-methyl-D-aspartate (NMDA) receptors, α-amino-3-hydroxy-5-methyl-4-isoxazolepropionic acid (AMPA) receptors, and kainate receptors.[5] The glutamatergic system and particularly the NMDA receptor play an important role in the neurobiology of major depressive disorder, and different glutamatergic targets have been considered as treatments for depression.[6,7] For example, antagonists of the NR2B subtype of the NMDA receptor are currently under development as potential antidepressant treatments; and traxoprodil, an NR2B antagonist, has demonstrated antidepressant effects in selective serotonin reuptake inhibitor (SSRI)-resistant patients.[7] Ketamine, the focus of this editorial, is an experimental antidepressant treatment which acts on NMDA receptors.[5]

## KETAMINE AS AN ANTIDEPRESSANT

Animal studies have shown that ketamine and other NMDA receptor antagonists have antidepressant effects in different animal models of depression.[8] About a decade ago, Berman *et al*.[9] described the first double-blind, placebo-controlled (crossover) study, which showed that an intravenous ketamine infusion (0.5 mg/kg) resulted in significant and rapid but short-lived antidepressant effects in seven patients with major depression.

Several years later, Zarate *et al*.[8] described the first randomized, double-blind, placebo-controlled (crossover) study of ketamine in 18 patients with treatment-refractory major depressive disorder. Ketamine (0.5 mg/kg) was administered as an intravenous infusion in normal saline across a 40-minute period; placebo comprised normal saline alone. There was significant antidepressant benefit within 110 minutes of the ketamine infusion. One day later, the response and remission rates to ketamine were 71% and 29%, respectively, and the antidepressant effect size was 1.5. However, only six (35%) patients maintained response for one week, by which time the effect size had dropped to 0.7. In contrast, the response rate to saline infusion was 0% after both one day and one week. Adverse effects with ketamine included perceptual disturbances, confusion, euphoria, dizziness, increased libido, and elevated blood pressure; these, however, lasted only for 1-2 hours.

Several case reports were subsequently published illustrating the efficacy of ketamine in medication-refractory patients. For example, Goforth and Holsinger[10] described a patient with severe, recurrent major depressive disorder who markedly improved within eight hours of receiving his first bitemporal electroconvulsive therapy (ECT) treatment under ketamine anesthesia. It is possible; however, that the dramatic response was to ECT and not necessarily to ketamine because such dramatic response to ECT has occasionally been described in literature. In another case report, Liebrenz *et al*.[11] described a 55-year-old male with treatment-resistant major depression and co morbid alcohol and benzodiazepine dependence, whose depression ratings dropped by >50% from the severely depressed into the mildly depressed range within two days of receiving an intravenous ketamine infusion of 0.5 mg/kg across 50 minutes. In this patient, the benefits of ketamine treatment were maintained for a week.

Other studies subsequently appeared. Price *et al*.[12] showed that suicide ratings dropped significantly within a day of treatment with ketamine (n=26); thrice-weekly ketamine infusions across a 12-day period were associated with sustained fall in suicidality scores (n=9). The authors considered that the attenuation of suicidality was likely to be a result of the antidepressant effect of the treatment rather than a specific antisuicidal effect. Aan het Rot[13] administered a single ketamine (0.5 mg/kg) infusion to 10 patients with treatment-refractory depression; a day later, MADRS scores in nine patients showed >50% attenuation. These nine patients received five more ketamine infusions across the next 10 days. At the end of the ketamine course, there was 85% attenuation in MADRS ratings in the sample. The patients were maintained drug-free; eight patients relapsed 6-45 (mean, 19) days after the last infusion. One patient remained antidepressant-free with minimal depressive symptoms for >3 months. In this study, three patients experienced significant but transient dissociative symptoms; other adverse effects during and after each ketamine infusion were generally mild.

Finally, in an open-label clinical trial of ketamine vs. propofol anesthesia in 31 medication-resistant depressed inpatients referred for ECT, Okamoto *et al*.[14] found that the speed of response and the magnitude of response were both greater with ketamine anesthesia at the end of a course of eight ECTs administered across four weeks. A limitation of this study is that the choice of anesthesia was based on patient preferences after informed consent; thus, an expectancy effect could have biased the outcomes.

*In summary*, intravenous ketamine infusion (0.5 mg/kg in normal saline, delivered across 40 min) is associated with dramatic antidepressant and antisuicidal effects in patients with major depression, including patients who are treatment-refractory. The benefits develop within 1-2 hours of the infusion, peak 1-2 days later, and persist for up to a week. Repeated infusion of ketamine is associated with maintained antidepressant effects for up to nearly two weeks; again, discontinuation of treatment is associated with relapse after an average of nearly three weeks. Adverse effects of ketamine infusion include confusion, dizziness, euphoria, perceptual disturbances, and increased blood pressure; these usually last for not more than 1-2 hours. Infusion of ketamine in sub anesthetic doses may therefore be a useful though temporary antidepressant measure in patients with refractory depression.

## THE POSSIBLE IMPORTANCE OF CHIRALITY

Ketamine is a chiral drug with R and S enantiomers. S-ketamine has greater affinity for the phencyclidine binding site on the NMDA receptor; S-ketamine is also three times more potent an anesthetic and two times more potent an analgesic than R-ketamine.[15] In equianalgesic doses, R-ketamine may be more psychotomimetic than S-ketamine.[16,17] Therefore, may S-ketamine be safer and more effective than R-ketamine in the treatment of depression?

Paul *et al*.[18] examined the possibility in two patients with treatment-resistant, recurrent major depression. One patient was a 51-year-old man and the other, a 58-year-old woman. Both were treated with racemic ketamine (0.5 mg/kg) and S-ketamine (0.25 mg/kg), delivered in 50-minute infusions. The two treatments were administered one week apart and in different order to the two patients.

The male subject did not respond to S-ketamine or racemic ketamine, administered in that order, a week apart. The female subject showed about 50% attenuation of Hamilton Depression Rating Scale (HAM-D) scores with both racemic ketamine and S-ketamine, administered in that order, a week apart. With both treatments, response peaked at 1-3 days and was lost by day 6. Both subjects experienced transient, mild perceptual disturbances with racemic ketamine but not S-ketamine. The male patient also experienced a crying episode with racemic ketamine. Neither subject experienced cardiovascular disturbances with either drug. This report therefore implies that S-ketamine (0.25 mg/kg) may be as effective an antidepressant as racemic ketamine (0.5 mg/kg) and may not induce perceptual or mood disturbances as does racemic ketamine.

It may be noted that, in this study, S-ketamine was administered in the dose of 0.25 mg/kg; that is, in the quantity that is contained in 0.5 mg/kg of the racemic mixture. As the effects of S-ketamine and racemic ketamine were similar, might the results of this report imply that R-ketamine has no contribution to the treatment of depression? The safety and efficacy advantage of S-ketamine over R-ketamine or racemic ketamine require to be assessed in formally designed randomized controlled trials.

## CONCLUDING OBSERVATIONS

Not all patients respond to ketamine. As variations in dose have not been studied so far, research is needed to determine whether higher or lower doses of the drug elicit antidepressant response in patients who fail to respond to the usual dose of 0.5 mg/kg. Another possibility in treatment failures is to consider repeated sessions of ketamine, such as at once-daily frequency, as is done in a course of electroconvulsive therapy. Strategies to maintain treatment response need to be identified for those who respond to the treatment; maintenance antidepressant pharmacotherapy or maintenance ketamine sessions are possibilities that spring to mind.

Finally, the expression of brain-derived neurotrophic factor (BDNF) triggers the neuroplastic changes that hypothetically represent the final common pathway of antidepressant action.[3] However, whereas both antidepressant drugs and ECT increase BDNF levels, ketamine does not.[19] This suggests that the dramatic antidepressant effects of ketamine may recruit other mechanisms; possibilities were discussed by Machado-Vieira *et al*.[20] Among other explanations, they suggested that AMPA to NMDA receptor throughput may represent a convergent mechanism for the rapid antidepressant action of the drug.
